# Reply to 'No substantial long-term bias in the Cenozoic benthic foraminifera oxygen-isotope record'

**DOI:** 10.1038/s41467-018-05304-3

**Published:** 2018-07-23

**Authors:** S. Bernard, D. Daval, P. Ackerer, S. Pont, A. Meibom

**Affiliations:** 1 Muséum National d’Histoire Naturelle, Sorbonne Université, CNRS UMR 7590, IRD, Institut de Minéralogie, de Physique des Matériaux et de Cosmochimie, Paris, 75005 France; 2 LHyGeS, CNRS UMR 7517, Université de Strasbourg/EOST, 1 Rue Blessig, Strasbourg, 67084 France; 3 Laboratory for Biological Geochemistry, School of Architecture, Civil and Environmental Engineering, École Polytechnique Fédérale de Lausanne (EPFL), Lausanne, 1015 Switzerland; 4 Center for Advanced Surface Analysis, Institute of Earth Sciences, University of Lausanne, Lausanne, 1015 Switzerland

## Introduction

Geochemical studies of biogenic calcite in the marine sediment record have contributed enormously to the understanding of Earth’s climate evolution. In particular, the oxygen-isotope compositions of fossil planktonic and benthic foraminifera tests are used as proxies for surface- and deep-ocean paleotemperatures, respectively^1,2^. Interpreted at face value, these compositions indicate Eocene deep-ocean and high-latitude surface ocean temperature in the range of 10–15 °C, and deep-ocean even warmer during the Cretaceous^1,2^. However, we demonstrated that oxygen-isotope re-equilibration through solid-state diffusion can create large errors in ocean paleoenvironmental reconstructions, even under the close-to-ambient pressure and temperature conditions characterizing shallow sediment burial^3^. Evans et al.^4^ question this conclusion, arguing that there is “No substantial long-term bias in the Cenozoic benthic foraminifera oxygen-isotope record”.

Evans et al.^4^ defend the idea of an extremely warm early Cenozoic (~50 Ma) by referring to fossils of “cold-blooded reptiles living in the Arctic and Antarctic circles”. We note that the interpretation of the polar fossil record (which is restricted to a few localities^5,6^) is based on the fragile assumption that these animals had the same physiology and thermal tolerance as presumed living relatives. However, very little (if anything) is known about the metabolism, the hibernation strategies, or the migration potential of these fossil species. For instance, recently discovered fossils of polar dinosaurs are interpreted to have lived under climatic conditions far from tropical^7,8^. In addition, a feature of the high-arctic world that has not changed since the Cretaeous is polar night^6^: nonmigrating polar species must have had a specific physiology that allowed them to withstand 3–4 months of total darkness with zero to subzero temperatures. These polar fossils may not be perfect analogs of presumed living relatives.

Evans et al.^4^ state that “Alternative quantitative Eocene proxy data from the high-latitude surface ocean can be used as an independent means of assessing the benthic foraminifera δ^18^O record, as the temperature of the deep ocean cannot be greatly decoupled from mean annual sea surface temperature in the region(s) of deep water formation due to the thermal inertia of water.” Yet, the thermohaline circulation likely varied in the past. Most models predict a weakened (if not arrested) ocean thermohaline circulation under high atmospheric CO_2_ conditions^9–11^. High-latitude ocean surface waters may well have been largely decoupled from deeper waters.

It might be worth investigating the long-term stability of these alternative proxies. In fact, as highlighted by Evans et al.^4^, these proxies indicate a very weak latitudinal thermal gradient in the surface waters during the Eocene (even weaker than the gradient indicated by the oxygen-isotope composition of fossil planktonic foraminifera). Such a weak gradient requires latitudinal heat transport of impossibly high efficiency^12–14^. In contrast, we demonstrated that, corrected for burial-induced isotope re-equilibration, a temperature gradient between low- and high-latitude surface ocean waters consistent with state-of-the-art climate models is re-established for the foraminifera oxygen-isotope record of the late Cretaceous and Paleogene^3^.

**Fig. 1:**
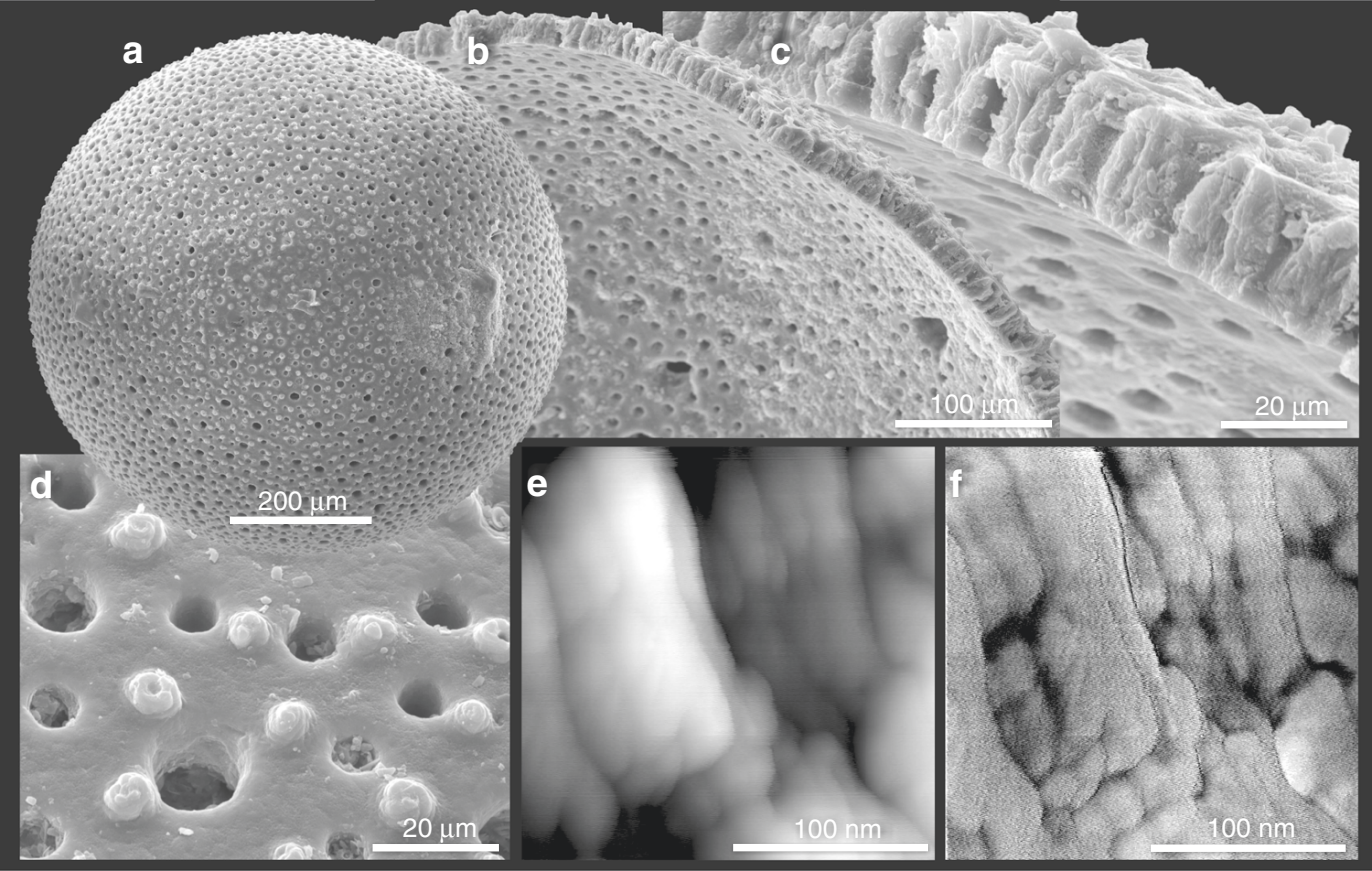
Images of a pristine planktonic foraminiferal test (*Orbulina universa*) from the macroscopic scale to the submicrometer-scale of the granular calcite “building blocks”. **a**–**d** Scanning electron microscopy (SEM) images of the outer surface exhibiting a large, porous surface area. **e**, **f** Atomic force microscopy (AFM) height (**e**) and phase (**f**) mode images. Courtesy of Prof. Stolarski

Pristine tests of foraminifera exhibit irregularly shaped calcite grains of only a few tens of nanometers (Fig. 1). As early as the 1950s, Urey et al.^15^ discussed the problem of preserving biogenic calcite oxygen-isotope records over geological time scales, specifically addressing resetting by diffusion. At that time, they wrongly assumed typical calcite grain sizes around 1 mm (they believed that bivalve shell calcite prisms were single crystals) and concluded that burial-induced isotope re-equilibration would be insignificant. We conducted numerical simulations conservatively assuming calcite grain sizes between 50 and 250 nm and demonstrated that isotopic re-equilibration of oxygen through diffusion can induce biases in paleotemperature reconstructions on time scales of 10^6^–10^7^ years. Of note, inserting a (conservative) grain size of 200 nm into the calculations by Urey et al.^15^ yields results very similar to ours. Because biogenic calcites are strikingly similar at microscales^16,17^, despite their great morphological diversity at the macroscale, all biocalcites are likely to be similarly sensitive to burial-induced re-equilibration processes.

Evans et al.^4^ show δ^18^O values from foraminifera of the same age retrieved from 11 different sites and states that “although the 11 sites span a range in maximum (i.e., present day) burial depth of between ~50 and 350 m, there is no visible offset between any individual site and the five-point running mean through all sites.” But this does not put into question our conclusions. The extent of diffusion is controlled by the temperature to which the foraminifera tests are exposed within the sediments. This temperature does not only depend on the burial depth, but also on the local thermal gradient. For instance, Eocene foraminifera tests collected from ODP sites 690 and 738 were buried below ~130 m of sediments with a thermal gradient of about 70 °C km^−1^, and below ~230 m of sediments with a thermal gradient of about 40 °C km^−1^, respectively^18–21^. In other words, these fossil tests were exposed to similar temperature conditions within the sediments; i.e., ~10–12 °C (which is, by the way, the temperature indicated by their present day oxygen-isotope compositions).

Of note, we investigated the impact of diffusion, but the much faster process of pseudomorphic coupled dissolution-reprecipitation^22,23^ also has the potential to bias paleotemperature reconstructions. It was recently demonstrated experimentally that isotope compositions of carbonates may change at room temperature over short timescales (<years), even after fluid–mineral chemical equilibrium has been attained^24,25^. According to Oelkers et al.^25^ “These observations are consistent with the hypothesis that mineral–fluid equilibrium is dynamic (i.e., dissolution and precipitation occur at equal, nonzero rates at equilibrium).” This process may impact elemental ratios as well.

We cannot follow Evans et al.^4^ when they state that secondary calcite precipitation “will result in calcite with a similar δ^18^Oc to the primary foraminiferal calcite”. In fact, because the water temperature is not the same everywhere in both the ocean and the sediment, secondary calcite precipitation that occurs within the sediments (increasingly so with increasing burial depth^26^) inevitably alters the bulk oxygen-isotope composition of fossil foraminifera tests^27,28^.

In any case, we fully agree that “a thorough understanding of diagenetic processes is essential to informative palaeoclimate reconstructions” and thank Evans et al.^4^ for this correspondence, which nourishes strong motivation for future investigations.

### Data availability

The datasets generated during and/or analyzed during the current study are available from the corresponding author on reasonable request.
